# Differential survival of 3rd stage larvae of *Contracaecum rudolphii* type B infecting common bream (*Abramis brama*) and common carp (*Cyprinus carpio*)

**DOI:** 10.1007/s00436-019-06441-4

**Published:** 2019-09-07

**Authors:** K. Molnár, C. Székely, F. Baska, T. Müller, S. Zuo, P. W. Kania, B. Nowak, K. Buchmann

**Affiliations:** 1 Institute for Veterinary Medical Research, Centre for Agricultural Research, Hungarian Academy of Sciences, Budapest, Hungary; 2 Department of Exotic Animal and Wildlife Medicine, University of Veterinary Medicine, Budapest, Hungary; 3 Department of Aquaculture, Szent István University, Gödöllő, Hungary; 4 Laboratory of Aquatic Pathobiology, Department of Veterinary and Animal Sciences, Faculty of Health and Medical Sciences, University of Copenhagen, Copenhagen, Denmark; 5 Institute of Marine and Antarctic Studies, University of Tasmania, Hobart, Tasmania Australia

**Keywords:** Susceptibility, Resistance, Bream, Carp, Nematodes

## Abstract

The main fish host reaction to an infection with third stage anisakid nematode larvae is a response in which host immune cells (macrophages, granulocytes, lymphocytes) in affected internal organs initially are attracted to the parasite whereafter fibroblasts may enclose the parasite forming granuloma. Generally, the reaction is non-lethal to the parasite which may survive for years in the fish host retaining infectivity to the final host. This may also apply for the anisakid nematode *Contracaecum rudolphii* (having the adult stage in cormorants, using copepods as first intermediate/paratenic host and zooplankton feeding fish as paratenic hosts). The present study has shown that most *Contracaecum rudolphii* larvae survive in bream (*Abramis brama*) (from Lake Balaton, Hungary) whereas the majority of the nematode larvae die in *Cyprinus carpio* (from Lake Hévíz, directly connected to Lake Balaton). Both cyprinid host species interacted with the nematode larvae through establishing a marked cellular encapsulation around them but with different effects. The differential survival in common carp and bream may theoretically be explained by ecological factors, such as the environmental temperature which either directly or indirectly affect the development of nematode larvae, and/or intrinsic host factors, such as differential immune responses and host genetics.

## Introduction

Third stage larvae of the anisakid nematode *Contracaecum rudolphii* Hartwich, 1964, are commonly occurring parasites in a range of fish species (mainly cyprinids) (Moravec 1994) in areas where the final hosts (fish-eating birds such as cormorants) are found. Although four other congeneric species, *C. microcephalum* (Rudolphi, 1809), *C. micropapillatum* (Stossich, 1890), *C. osculatum* (Rudolphi, 1802), and *C. ovale* (Linstow, 1907), occur in Europe, *C. rudolphii* seems to occur worldwide—from Europe to Australia (Shamsi et al. 2011). It has been indicated that *C. rudolphii* is a species complex—consisting of at least two strains, *C. rudolphii* A and *C. rudolphii* B (Mattiucci et al. 2002; D’Amelio et al. 2007; Zhu et al. 2007; Farjallah et al. 2008; Szostakowska and Fagerholm 2007, 2012). Experimental studies have elucidated the complete life cycle. Parasite eggs released from adult female worms in cormorants are expelled into the aquatic habitat with host feces. Following embryonation, the eggs hatch and the released larvae are ingested by copepods which serve as the first intermediate host. Zooplankton-feeding fish get infected when ingesting copepods carrying nematode larvae whereafter they serve as paratenic hosts until fish-eating birds, such as cormorants, ingest the fish. Finally, in the bird gastro-intestinal tract, the third stage larvae moult twice and obtain sexual maturity (Mozgovoy et al. 1968; Dubinin 1949; Dziekońska-Rynko and Rokicki 2007, 2008; Moravec 2009). Third stage larvae of *Contracaecum* spp. accumulate mostly in the mesentery and the intestinal serosa of fish but may also reside in the intestinal wall and liver. The genus *Contracaecum* has a worldwide distribution and includes a range of species which are commonly found in fish serving as paratenic hosts and employ warm-blooded animals (birds, pinnipeds) as final hosts (Mattiucci and Nascetti 2008; Aydogdu et al. 2011; Waicheim et al. 2014; Corrêa et al. 2015; Tavakol et al. 2015; Dezfuli et al. 2016; Zuo et al. 2018). When teleosts are infected by anisakid nematode larvae, they often react by enclosing the worm in a layer of host cells forming a granuloma (Buchmann 2012; Santoro et al. 2013; Corrêa et al. 2015; Dezfuli et al. 2016). In order to stay alive and be potentially infective to the final host, the parasite must inactivate the immune responses of the fish host. In addition, the development of the parasite in the host may be influenced by environmental factors, such as temperature. In this report, we describe an elevated survival of *C. rudolphi* larvae in a common bream (*Abramis brama* L.), compared with a low survival rate of the same parasite species in a common carp (*Cyprinus carpio* L).

## Materials and methods

### Ethical statement

All experiments and handling of fish in the present study were conducted according to the animal welfare guidelines and recommendations (permission number PEI/001/1002-13/2015) under the Veterinary Medical Research Institute, Hungarian Academy of Sciences, Budapest, Hungary.

### Study area

Fish were obtained from (1) Lake Balaton which is one of the largest shallow lakes in Europe and populated by more than 50 fish species (Daday 1897; Pintér 1989) of which the cyprinid common bream (*Abramis brama* L.) is the dominating species and (2) from the connected open thermal lake Hévíz, which is a biologically active natural thermal spa lake near Keszthely, Hungary. The lake is recognized as a nature conservation region covering an area of 4.44 ha containing hydrogen-carbonated water with a content of calcium and magnesium. During summer periods, the temperature of the lake Hévíz may reach 35–39 °C near the surface and does not fall below 26–29 °C even in the coldest winter. The water temperature in Lake Balaton varies from around 4–7 °C during winter time to 24–28 °C during summer.

### Fish

#### Bream

A total of 362 large bream (3 to 6 years old) and a total of 41 small bream were examined. The smaller-sized bream (10 to 20 cm in length) were collected from different parts of the Lake Balaton with a seine, while some larger specimens (20–40 cm in length) were collected at the drainage ditch of Lake Balaton (city Siófok) run by the Balaton Lake Fisheries Company during the draining period in late autumn and early spring.

#### Common carp

A total of 19 specimens of wild common carp were collected in the water of the Hévíz Lake by angling in 2017 and 2018 when 4 to 6 specimens were collected in different seasons (April, May, September, and November). These slowly growing 8–9-year-old carp specimens had achieved a 23–29-cm body size (Varga et al. 2013). The wild common carp is enlisted on the IUCN Red List as ‘vulnerable’ but the Danube subpopulation (*Cyprinus carpio morpha hungaricus* Heckel, 1836) is listed as ‘critically endangered’ fish (http://www.iucnredlist.org).

### Parasitological examination

Live fish were transported to the laboratory in oxygenated plastic bags, subsequently transferred to and kept in laboratory aquaria, and subjected to complete parasitological dissection within 3 days post-arrival. All examinations were carried out using freshly killed fish. The fish were anaesthetized by immersion into the water with 20 ppm clove oil Javaheri et al. 2012); whereafter, fish were euthanized by a blow to the head. In all cases, a specific search for nematode larvae was performed in all organs. The gut was divided into 5 equal parts numbered from anterior to posterior. Organs removed from the fish were studied under a dissection microscope followed by a more detailed study with a compound microscope. The number of *Contracaecum* nematode larvae was recorded and fixed for further identification (light microscopy, histology) and a subsample of 15 larvae was conserved for molecular identification. The intensity of infection was recorded by counting larval nodules in the abdominal cavity whereafter the mean intensity and range were noted. Viability of larvae was tested by mechanically opening the surrounding connective tissue enclosement and recording any larval motility. The infection level was calculated as prevalence (percentage of investigated fish infected) and mean intensity (mean number of parasites per infected fish).

### Histology

When nematode larvae were observed in the abdominal cavity associated with various organs (including the gut and peritoneum), the worms with enclosing tissues were fixed in 10% buffered formalin for 10 to 20 days or in Bouin’s solution for 24 h. Subsequently, specimens were dehydrated through graded series of ethanol and embedded in paraffin wax whereafter sections (4–5 μm) were cut and stained with haematoxylin and eosin. Preparations were studied using Nomarski differential interference contrast with an Olympus BH2 microscope and photographed with an Olympus DP 20 digital camera.

### Identification of larvae

Larvae collected from the mesentery and the intestine were studied under a coverslip and morphometrically identified according to Moravec (1994). Molecular confirmation was done on a subsample of 15 larvae using PCR and subsequent sequencing of ribosomal DNA (18S, 5.8S, 28S, ITS1, and ITS2) as outlined by Zuo et al. (2018). The middle part of the nematode larva was cut out aseptically and incubated in 100 μl lysis buffer (Tween 20 (0.45%), Proteinase K (60 μl/ml), 10 mM Tris, and 1 mM EDTA at 55 °C, 450 rpm) in the Eppendorf Thermomixer Comfort (Eppendorf AG, Hamburg, Germany). Incubation time varied but continued until complete digestion. Proteinase was then deactivated at 95 °C for 10 min and the lysate was used for PCR amplification. PCR was performed in a Biometra T3 thermocycler (Fisher Scientific) using 60-μl reaction volumes. The reaction mixtures consisted of 6 μl lysate as a template, 1 unit of BioTaq DNA polymerase (DNA-Technology), 1 mM dNTP, 1.5 mM MgCl_2_, and 1 μM of the two primers. In order to amplify the ITS region, the primers NC5 (5′GTA GGT GAACCT GCG GAA GGA TCA TT-3′) and NC2 (5′TTA GTT TCT TTTCCT CCG CT-3′) were used as forward and reverse primer, respectively (Zhu et al. 2007). PCR conditions were 2 min of pre-denaturation at 94 °C followed by 36 cycles of denaturation at 94 °C for 30 s, annealing at 53 °C for 30 s, elongation at 72 °C for 1 min 15 s. Finally, a post-elongation step was performed at 72 °C for 7 min. Products were analysed by 2% ethidium bromide–stained agarose gels. PCR products were purified using Illustra GF PCR and Gel Band Purification kit (GE Healthcare, cat. no. 28-9034-71) according to the manufacturer’s instructions prior to sequencing at Macrogen Inc. (South Korea). Analyses of sequences were performed using the software CLC Main Workbench v.7.9.1 (Qiagen, Denmark) and confirmed by BLAST® analysis (Basic Local Alignment Search Tool) at GenBank (www.ncbi.nlm.nih.gov/BLAST).

## Results

### Identification

The morphometric analysis of third-stage larvae and the molecular analysis of ITS sequences obtained from PCR and sequencing showed that the larvae recovered in the present study belong to the species *Contracaecum rudolphii*. Out of 15 nematodes, 12 gave a PCR product. Ribosomal DNA from a total of 7 larvae from bream and 5 from carp were sequenced and the BLAST analysis showed 100% similarity to *C. rudolphii* B, GenBank sequence FJ467618 with one exception (one larva from bream with one substitution at position 411). The recovered sequences (length 972 bp) with 100% similarity were uploaded on GenBank with accession numbers MH778106–12 for larvae in bream with exception of the sequence with substitution C411 to S411 (S = G and C) having an accession number MH778107. Sequences for larvae in carp, all with 100% similarity to FJ467618 have accession numbers MH778113–17. All larval sequences showed affiliation with *C. rudolphii* type B (GenBank accession number DQ316968) (Szostakowska and Fagerholm 2007, 2012).

### Infection levels

**Fig. 1:**
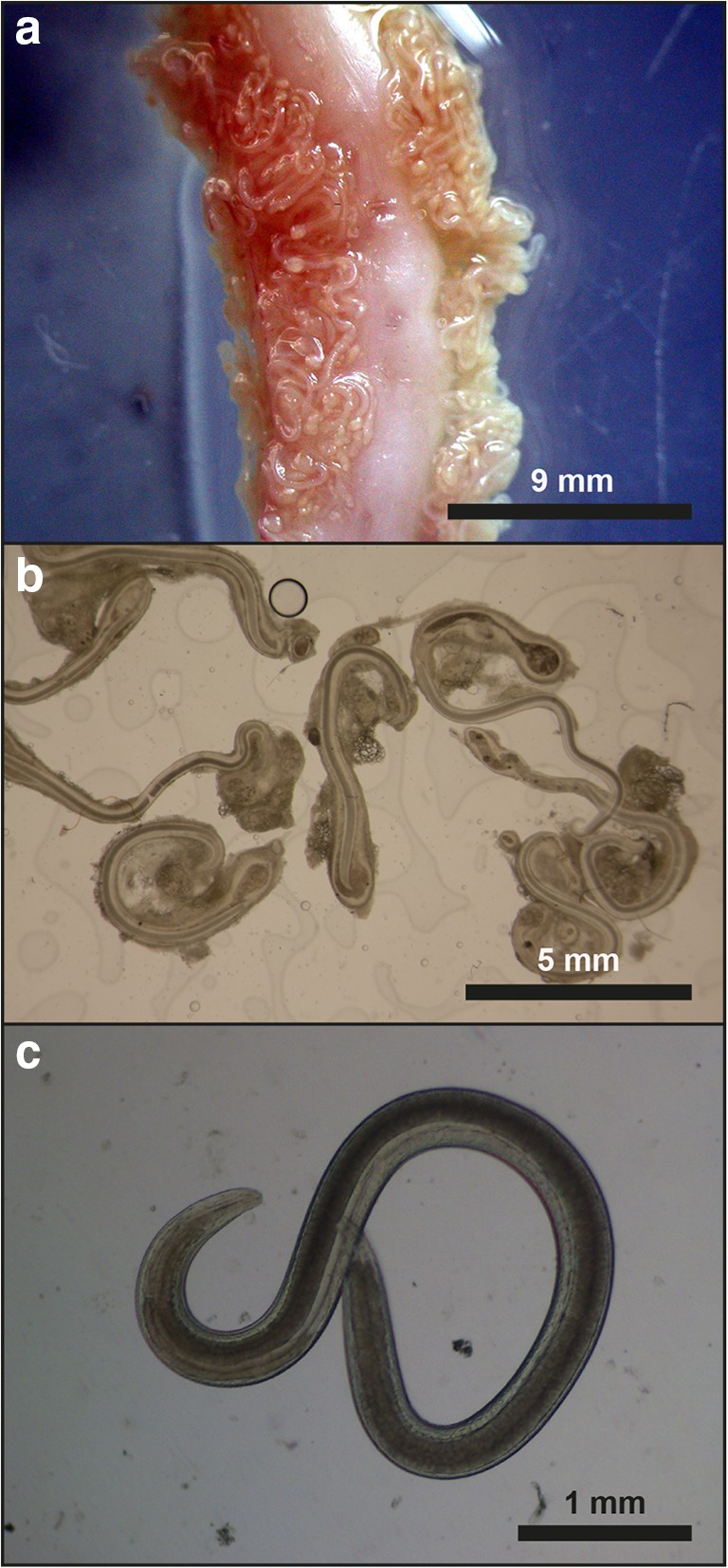
**a** Fourth intestinal segment of the common bream including the peritoneum heavily infected with *Contracaecum rudolphii* larvae. **b***Contracaecum rudolphii* larvae from the body cavity of a common bream. Live larvae are enclosed by cellular host reactions. Fresh mount. **c** Motile *Contracaecum rudolphii* larva removed from its cellular enclosure in common bream. Fresh mount

A total of 331 out of the 362 dissected common breams (prevalence 91%) harboured *Contracaecum* larvae. Of the 1- and 2-year-old bream, only two specimens (prevalence 5%) harboured *Contracaecum* larvae. Granulomas were mainly located in the mesentery of the fourth segment of the gut (Fig. 1a). In that segment, nodules were mostly found in the mesentery and the serous membranes surrounding the gut, but some were also found in the intestinal wall as well. The number of granulomas in individual bream hosts varied between 17 and 150 (mean intensity 74, SD ± 35.6) parasites per infected fish. Most nematode larvae (> 90%) in bream had preserved their viability despite being markedly enclosed by cellular reactions (Fig. 1b). There was no observable difference in the number of living and dead larvae during the cool and warm period of the year. Freshly released larvae demonstrated motility during and following release (Fig. 1c). A total of 13 fish (prevalence 68%, mean intensity 36.2) out of the 19 common carp specimens examined were infected with *Contracaecum* nematode larvae or remnants thereof, but live larvae (total 156) were detected in only 5 fish (prevalence 26.3%). In this case, also, dead and damaged larvae (total 157) were found. In five carp, merely dead decaying larvae (total 158, mean intensity 31.6) and amorphous material were found. In three fish, only nodules containing amorphous material and the shrunken cuticle of dead worms were recorded.

### Histopathological observations

**Fig. 2:**
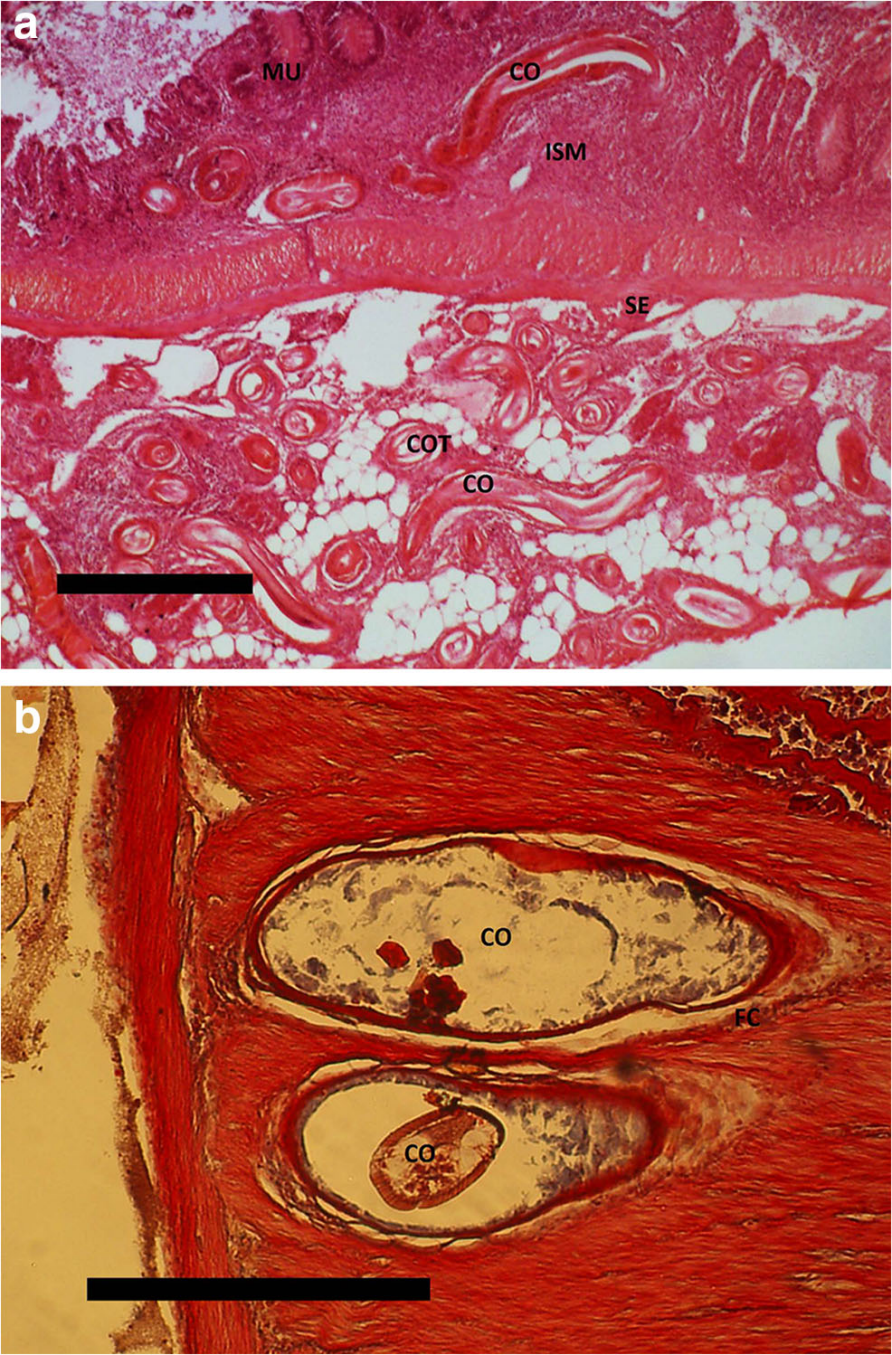
**a** Section of intestinal tissue with mucosa (MU), submucosa (ISM), and serosa (SE) of common bream. *Contracaecum rudolphii* larvae are seen in longitudinal section (CO) and transverse section (COT). H&E staining. Scale bar 250 μm. **b** Common carp (muscularis propria) infected by *Contracaecum rudolphii* larva (CO). Infection with decaying larva surrounded by a fibrous capsule (FC). H&E staining. Scale bar 250 μm

**Fig. 3:**
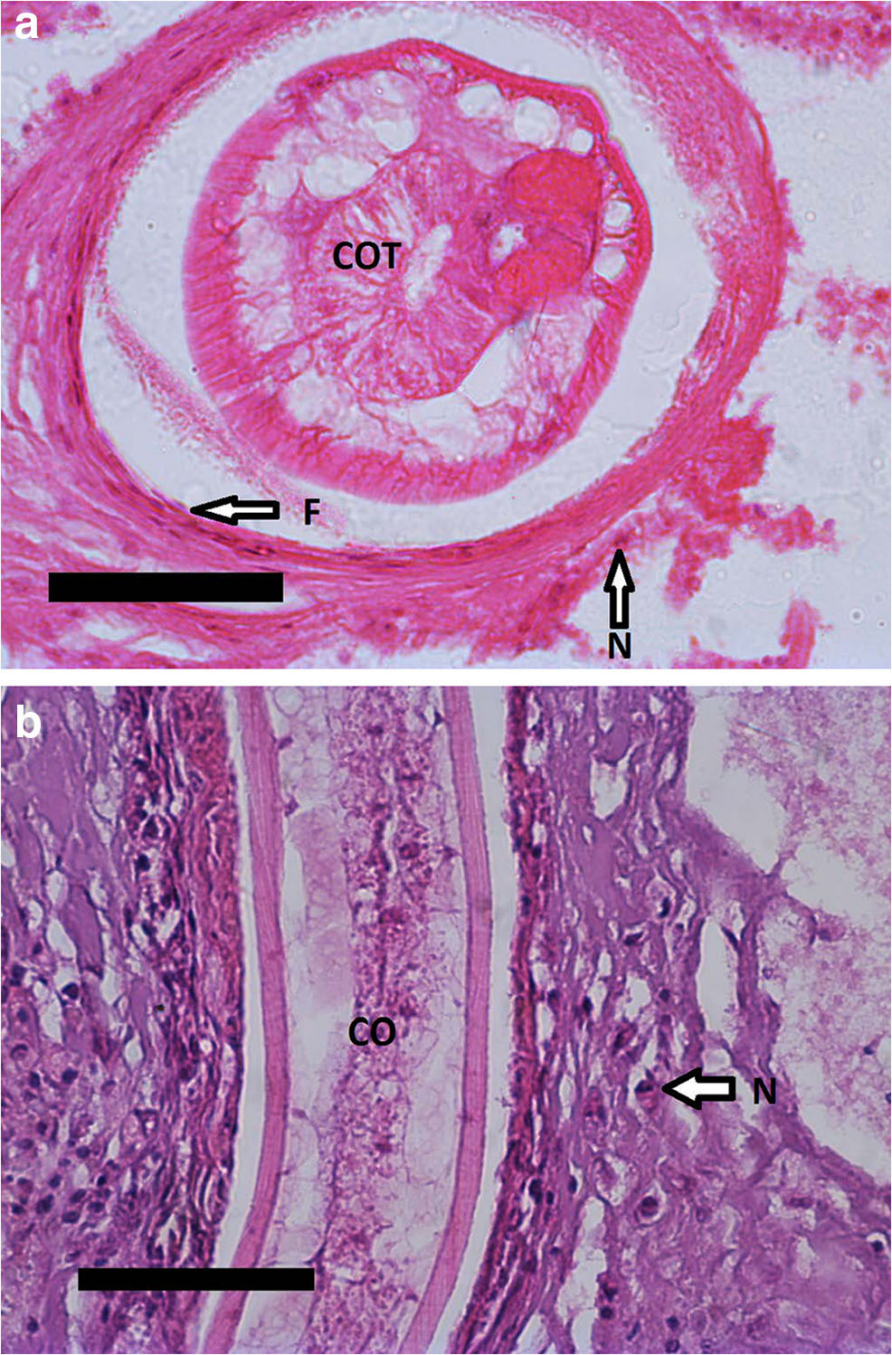
**a** Common bream infected by *Contracaecum rudolphii* larva (COT). Section of a fibrous capsule with fibroblasts (F) and some neutrophilic granulocytes (N). H&E staining. Scale bar 50 μm. **b** Common carp serosa with *Contracaecum rudolphii* larvae (CO) surrounded by a fibrous capsule. Neutrophilic granulocytes (N) are shown. H&E staining. Scale bar 50 μm

Parasitic granulomas containing *Contracaecum* larvae were located both in the peritoneum and in the intestinal wall of both bream and carp (Fig. 2a, b). Most granulomas in the bream peritoneum contained live larvae, enclosed by 1 to 2 layers of host cells (macrophages, fibroblasts, neutrophilic granulocytes) (Fig. 3a). *Contracaecum* larvae were found both in the peritoneum and in the intestinal wall of carp, but in a few fish, some larvae invaded the liver tissue. More progressed (chronic) stages showed capsules with one to seven cell layers. In the mesentery, live larvae surrounded by a thin layer of connective tissue were found together with decaying larvae surrounded by a thick multi-layered connective tissue capsule (Fig. 2b). These progressed reactions with several cell layers were dominated by fibroblasts but with the presence of lymphocytes, macrophages, and neutrophilic granulocytes (Fig. 3b).

## Discussion

The parasite fauna in Lake Balaton fishes has been relatively well studied, and several nematode species have been reported from local fish (Molnár and Székely 1995; Molnár et al. 2001, 2002; Székely et al. 2010). Among these, *C. rudolphii* is commonly found as third-stage larva in bream as the fish serves as paratenic host with the cormorant as final host. The occurrence of the common carp in Lake Balaton is exclusively a result of stocking of older carp from fish farms, and they are therefore not infected with *C. rudolphii* in this lake—presumably because these older fish do not feed on copepods. However, in the Hévíz Lake, located next to Lake Balaton, a common carp population is reproducing and carp fry obtain infection with the same parasite species by feeding on infected copepods. This allowed us to compare the host reactions of bream and carp with *C. rudolphii*, and it is noteworthy that both fish species interact with the nematode larvae by an encapsulation process, but the nematode larvae showed different survival. Most *C. rudolphii* larvae in bream were recovered as live and motile specimens whereas the majority of isolated larvae in common carp were dead. Nematode larvae from various taxonomic lineages including anisakid nematodes, which are using warm-blooded animals as final hosts, must remain alive and infective in the fish-transport host in order to reach the adult and reproducing stage in the final host. Otherwise, successful completion of the life cycle and survival of the species in the habitat will be blocked. Different fish species, serving as paratenic hosts, may contribute differently to the life cycle depending on their ability to eliminate the nematode larvae following infection. Thus, a cellular reaction may not always lead to elimination of the invader. Initially, various immune cells are attracted to the site of larval entry, and subsequently, fibroblasts may enclose the live worm in a dense host layer (Buchmann 2012). The larva may then in some fish survive for years in such a hypobiotic state until the final host ingests the infected fish and the larva moults twice to reach the adult stage. Previous studies by Dezfuli et al. (2016) demonstrated a marked cellular response in *Anguilla anguilla* L. against the *C. rudolphii* larvae. The larva invaded the intestinal wall and the peritoneum, and elicited granuloma formation with inclusion of macrophages, mast cells, and fibroblasts, but the effect on the parasite survival was not addressed. In the present study, we investigated the host reactions of two cyprinid fish species to third-stage larvae of *C. rudolphii* and present evidence for a differential survival of the nematode larvae. Bream *Abramis brama* enclosed the nematode larvae by several layers of host cells, but the majority of the parasites resisted the reaction and stayed alive. We showed that common carp *Cyprinus carpio* also reacted with a cellular response and that a considerable part of the invading larvae was found dead. The present study was based on fish sampled in two different lakes where the temperature differed. Therefore, it cannot be excluded that this environmental factor directly or indirectly may have influenced the outcome of the present study. It is known that the teleost immune response is affected by temperature (Raida and Buchmann 2007) and a higher temperature in Lake Hévíz may at least partly accelerate the antiparasitic response. Therefore, controlled infection studies should be done to elucidate this possibility.

In Lake Balaton, a corresponding interactive system was previously reported by Székely (1995) demonstrating a significant difference in reactions of various paratenic fish host species to *Anguillicoloides crassus* (Kuwahara, Niimi & Itagati, 1974) larvae. Even when comparing closely related cyprinid species—kept in the same habitat with the same temperature—the survival of *A. crassus* larvae in host tissues differed markedly. Our comparative observations suggest that differential immune responses in different fish hosts may explain at least part of the different infection levels found in bream and carp, but the extent to which these are influenced by temperature must be elucidated. Further, improved methods for recovering *C. rudolphii* larvae from carp tissues as recommended by Shamsi and Suthar (2016) may provide a more precise estimation of infection levels and thereby add to our knowledge on pathogenic effects on host physiology. We therefore advocate for future controlled experimental infection studies on common carp and common bream using infective *C. rudolphii* larvae in order to confirm this hypothesis.
